# Adverse Events during Prone Positioning of Patients with COVID-19 during a Surge in Hospitalizations—Results of an Observational Study

**DOI:** 10.3390/nursrep14030132

**Published:** 2024-07-19

**Authors:** Nataša Radovanović, Mateja Krajnc, Mario Gorenjak, Alenka Strdin Košir, Andrej Markota

**Affiliations:** 1 Infectious Diseases Intensive Care Unit, University Medical Centre Maribor, 2000 Maribor, Slovenia; 2 Medical Intensive Care Unit, University Medical Centre Maribor, 2000 Maribor, Slovenia; 3 Centre for Human Genetics and Pharmacogenomics, Faculty of Medicine, University of Maribor, 2000 Maribor, Slovenia; mario.gorenjak@um.si

**Keywords:** mechanical ventilation, prone positioning, adverse events, COVID-19, removal of catheters and tubes, pressure ulcers

## Abstract

This study aimed to determine the prevalence of adverse events in mechanically ventilated adults with COVID-19 who have undergone prone positioning. A total of 100 patients were included retrospectively; 60% were males, the mean age was 64.8 ± 9.1 years, and hospital mortality was 47%. In all, we recorded 118 removals of catheters and tubes in 66 patients; 29.6% were removals of a nasogastric tube, 18.6% of an arterial line, 14.4% of a urinary catheter, and 12.7% of a central venous catheter. Reintubation or repositioning of a tracheotomy tube was required in 19 patients (16.1%), and cardiopulmonary resuscitation in 2 patients (1.7%). We recorded a total of 184 pressure ulcers in 79 patients (on anterior face in 38.5%, anterior thorax in 23.3% and any extremity anteriorly in 15.2%). We observed that body weight (*p* = 0.021; β = 0.09 (CI95: 0.01–0.17)) and the cumulative duration of prone positioning (*p* = 0.005; β = 0.06 (CI95: 0.02–0.11)) were independently associated with the occurrence of any adverse event. The use of prone positioning in our setting was associated with a greater number of adverse events than previously reported. Body weight and cumulative duration of prone positioning were associated with the occurrence of adverse events; however, other factors during a COVID-19 surge, such as working conditions, staffing, and staff education, could also have contributed to a high prevalence of adverse events.

## 1. Introduction

Coronavirus disease (COVID-19), caused by the Severe acute respiratory syndrome Coronavirus 2 (SARS-CoV-2), frequently leads to the development of acute respiratory distress syndrome (ARDS) [1]. ARDS is associated with high mortality even when mechanical ventilation and other critical care supportive measures are utilized. During the initial pandemic waves, up to one-third of patients with COVID-19 progressed to ARDS. Most of those patients required mechanical ventilation and critical care treatment, significantly burdening healthcare systems. Prone positioning (PP) has emerged as a critical intervention for improving oxygenation and survival in mechanically ventilated patients with ARDS [1,2]. Shifting the body position from a supine to prone position helps reduce pleural pressure gradients from dependent to nondependent lung regions resulting in increased functional residual capacity, improved matching of ventilation to perfusion, and, in some cases, improved lung drainage. Evidence supports the efficacy of PP in improving outcomes for ARDS patients both in pre-pandemic settings and during the COVID-19 pandemic [1,2]. Despite its benefits, PP is associated with numerous adverse events [3,4], such as accidental extubation [2], unplanned removal of central venous catheters (CVCs), arterial lines, and thoracostomy tubes [3,5,6], hemodynamic instability [5,6], pneumothorax, cardiac arrest [3], facial, airway, and chest edema, conjunctival hemorrhage, endotracheal obstruction, and brachial plexus injury among others [2,5,6]. The most common complication of PP is the development of pressure ulcers of the anterior body [3,4,7,8]. We aimed to determine the prevalence of adverse events in mechanically ventilated adults with COVID-19 who have undergone prone positioning.

## 2. Materials and Methods

### 2.1. Study Design and Setting

We performed a retrospective observational study on patients who were treated in the COVID intensive care unit (ICU) at a tertiary hospital serving a population of approximately 800,000 inhabitants. We included adult patients admitted to the COVID ICU from the hospital emergency department, hospital COVID department, or other hospital departments or were patients with COVID-19 from other regions in case of COVID ICU bed shortages in other hospitals. The 56-bed COVID ICU was located in converted hospital departments (e.g., cardiac surgery, neurosurgery, and psychiatry departments) for the purpose of treating a large number of patients who required mechanical ventilation. The COVID ICU functioned as a separate hospital department, but it was organized into 3 units with staff allocated to a distinctive unit, and inter-unit staff changeovers were kept to minimum. Approximately 1/3 of staff were permanently stationed in the COVID ICU during the pandemic waves, while the remaining staff rotated in and out of the COVID ICU typically every 3–6 months. Each unit was provisioned with similar or identical equipment to streamline supply logistics. Overall, the COVID ICU was staffed in all by 277 nurses, 36 doctors, and 21 physiotherapists, with about two-thirds of the nurses and half of the doctors being recruited to the COVID ICU from non-ICU departments.

Institutional ethics committee approval was obtained (No. 27/22), and the need for informed consent was waived due to the retrospective nature of the study.

### 2.2. Study Patients and Interventions

We included adult (>18 years) mechanically ventilated patients with COVID-19 who were treated with PP during a COVID-19 surge in the period between 1st September 2021 and 31st March 2022 (a 7-month period). Data were collected from medical charts and electronic medical records, including data on age and gender, weight and height, outcome of intensive care and hospital treatment, and data on adverse events related to PP. Temperature and therapeutic charts were paper-based, while laboratory, radiology, and microbiology reports were electronic. Removals of catheters, tubes, and drains were noted on the temperature and therapeutic charts on the day of the removal, and the reason for the removal was noted in the nursing and physicians’ notes. Data extraction was performed retrospectively from September 2022 to January 2023 by individual exploration of source data by researcher NR. Removals of catheters and tubes were defined as unplanned if a removal was noted during a PP session or within 24 h after the patients was supinated, unless explicitly noted in medical documents that the removal was pre-planned (e.g., removal of a pleural drainage tube after the evacuation of pleural effusion). Other adverse events were noted at the discretion of researcher NR (e.g., brachial plexus injuries, corneal injuries, etc.) and were not explicitly predefined.

Patients were pronated as per the treating physician. The usual departmental strategy was to maintain PP for a period of around 24 h regardless of the time of the initiation of PP. After around 24 h, the patient was reassessed, supinated, and, afterwards, pronated again as per the treating physician. PP was performed by a team of 4 nurses (2 per each side) and a physician. Usually, the “swimmer” position was used—i.e., when the head was rotated to the left; then, the left arm was extended to around 90° in the shoulder and flexed to around 90° in the elbow, and the left leg was flexed to around 90°in the hip and 90° in the knee, while the right arm was placed alongside the body, and the right leg was extended, and vice versa when the head was rotated to the right. The position of the head, arms, and legs was changed once per shift (every 8 h) because of the lack of healthcare workers during the pandemic waves. During the PP sessions, patients were deeply sedated and neuromuscular blocking agents were used as a lung protective ventilation strategy with target tidal volumes < 8 mL/kg, plateau pressure < 30 cm H_2_O, and driving pressure < 12 cm H_2_O.

Anti-decubitus mattresses (Apex domus 2 system, Wellell Inc., New Taipei City, Taiwan) were used in all patients to prevent pressure ulcers. Pillows (normal hospital pillows, GentleTouch Support pillows, Mizuho OSI, Union City, CA, USA, or SleepAngel Pillow, Gabriel Scientific OÜ, Pärnumaa, Estonia, as per availability) were usually placed under the head, and usually, a pillow (SleepAngel Positioner, Gabriel Scientific OÜ, Pärnumaa, Estonia) was placed below the chest and the pelvis. Before PP, the exposed parts of the anterior body where pressure ulcer formation could be anticipated (usually, the forehead, shoulders, chest, knees, and ankles) were protected with Mepilex polyurethane wound dressing (Mölnlycke Health Care AB, Gothenburg, Sweden).

Pressure ulcers were graded using the European Pressure Ulcer Advisory Panel (EPUAP) scale. The removal of central venous catheters, arterial catheters, drainage tubes, endotracheal tubes, or other tubes was defined as any unplanned removal during PP session or within 24 h after subsequent supination. One session of PP was defined as unbroken PP without supination—if the patient was supinated and re-pronated, that was a second (third, …) PP session.

### 2.3. Statistical Analysis

Data were analyzed using R 4.2.1 programming environment (R Core Team 2020, Vienna, Austria). Statistical differences in continuous variables between groups were determined using the Mann–Whitney U-Test after the Kolmogorov–Smirnov test of normality. The relationship between two continuous variables was assessed using Spearman’s correlation after the Kolmogorov–Smirnov test of normality. Statistical differences between two dichotomous nominal categorical variables were determined using Fisher’s exact test. Regressions were carried out using generalized linear models in order to correct for covariates or to estimate the independent impact of prediction variables. Statistically significant difference was considered at *p* < 0.05. Validation was performed using machine learning random forest algorithm using randomForest 4.6–14 R package in probability mode [9]. Recursive future elimination was performed using e1071 R package and msvmRFE implementation [10]. A graphical representation of obtained machine learning probabilities was constructed using receiver operating characteristic curves using R package pROC version 4.2.1 [11].

## 3. Results

In 100 mechanically ventilated adult patients with COVID-19 who required PP, we observed, in all, 118 removals of catheters, tubes, and drains and a development of 184 pressure ulcers during a total of 228 PP sessions. Nasogastric tube removals were most common, but removals of vascular, endotracheal, and tracheal tubes were also observed. Facial and thoracic pressure ulcers were most common. Increasing weight, greater cumulative duration of PP, higher age and height, female gender, and increasing number of PP sessions were associated with the development of any adverse event during PP.

### 3.1. Basic Demographic Data

During the study period, 463 patients infected with COVID-19 were hospitalized in the COVID ICU, of which 277 patients (59.8%) were mechanically ventilated, and 100 patients were treated with PP (21.6%). A total of 228 PP sessions were performed. The basic characteristics of the patients, including survival data, are presented in Table 1.

### 3.2. Removal of Vascular Catheters, Endotracheal and Tracheal Tubes, and Other Drainage Tubes and Catheters during Prone Positioning

During PP and within 24 h after supination, we recorded 118 removals of catheters and tubes in 66 patients, namely 15 cases (12.7%) of the removal of a central venous catheter, 22 cases (18.6%) of arterial line removal, 35 cases (29.6%) of nasogastric tube removal, 17 cases (14.4%) of urinary catheter removal, and 19 cases (16.1%) of reintubation or the repositioning of the tracheotomy tube. Additionally, we recorded 1 case (0.8%) of brachial nerve injury requiring follow-up by a neurologist and 2 cases (1.7%) of upper respiratory tract bleeding requiring follow-up by an otorhinolaryngologist. Initiation of cardiopulmonary resuscitation in the prone position was required in 2 cases (1.7%) (Table 2). A post hoc sample size calculation was performed yielding a sample size of 78 patients with 95% confidence level and 5% margin of error.

### 3.3. Pressure Ulcers Associated with Prone Positioning

We recorded a total of 184 pressure ulcers in 79 patients. We recorded 71 (38.5%) facial pressure ulcers, 43 (23.3%) anterior thorax pressure ulcers, 28 (15.2%) pressure ulcers on any extremity anteriorly, and 42 (22.8%) pressure ulcers on other locations of the body anteriorly. A post hoc sample size calculation revealed a sample size of 73 patients with 95% confidence level and 5% margin of error. Most of the pressure ulcers were EPUAP stage II. (91%), followed by stage III. (8.6%) (Table 3 and Table 4).

### 3.4. Additional Results

Additionally, we analyzed risk factors for the development of adverse events during PP. The cumulative duration of PP was longer in patients with any adverse event compared to patients without adverse events (76.7 ± 38.2 h vs. 37.3 ± 12.1 h, *p* = 4.3 × 10^−4^). Also, a higher number of PP sessions was associated with more adverse events (2.3 ± 1.3 sessions vs. 1.1 ± 0.3 sessions, *p* = 4.8 × 10^−4^) (Table 5). However, the duration of individual sessions was observed only for the second PP session (the duration of the first session was 24 h as per departmental strategy). The occurrence of a second PP session was associated with a higher EPUAP stage pressure ulcer on anterior face (*p* = 0.024; ρ = 0.290), on anterior thorax (*p* = 0.03; ρ = 0.279), on other locations of the body anteriorly (*p* = 0.006; ρ = 0.348), and pressure ulcer anywhere anteriorly (*p* = 0.006; ρ = 0.347). The occurrence of a third and fourth PP session did not correlate with a higher EPUAP stage pressure ulcer, most likely because of a lack of power.

Higher body weight and higher body mass index (BMI) were associated with a greater number of adverse events (87.7 ± 20.5 kg vs. 74.4 ± 12.2 kg, *p* = 0.015, and 29.4 ± 6.3 kg/m^2^ vs. 25 ± 2.3 kg/m^2^, *p* = 0.006, respectively).

**Figure 1:**
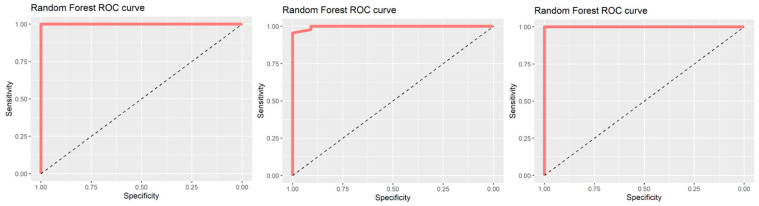
Random forest ROC curves. From left to right, area under the curve (AUC) for association between any adverse event and combined weight, cumulative duration of PP, age, height, sex, and number of PP sessions (**left panel**), weight and cumulative duration of PP (**middle panel**), and weight, cumulative duration of PP, and age (**right panel**).

Higher age and female gender were not associated with a greater number of adverse events (67.2 ± 6.4 vs. 64.5 ± 9.3, *p* = 0.593, and 36 (90.0%) vs. 52 (88.1%), *p* = 1.0, respectively). Moreover, observations from the second PP sessions were additionally assessed using generalized linear models in order to correct for BMI. It was observed that the duration of the second session still exhibits statistically significant association with a higher EPUAP stage pressure ulcer on anterior face (*p* = 0.012; β = 4.96) and shows a tendency for association with pressure ulcer on anterior thorax (*p* = 0.067; β = 2.61). Furthermore, we used machine learning algorithms in order to assess the relationship weights between any adverse event and body weight, cumulative duration of PP, age, height, sex, and number of sessions. Using recursive feature elimination algorithm, we ranked the aforementioned variables in the following manner from having the most to the least impact on any adverse event: Weight, cumulative duration of PP, age, height, sex, and number of PP sessions. All variables were further assessed using the random forest machine learning algorithm. All six variables together produced an area under the curve (AUC) of 1; the first two variables produced AUC of 0.99 (CI95: 0.99–1.00), and the first three variables produced AUC of 1, confirming the involvement of these variables in the development of adverse events during PP (Figure 1).

**Figure 2:**
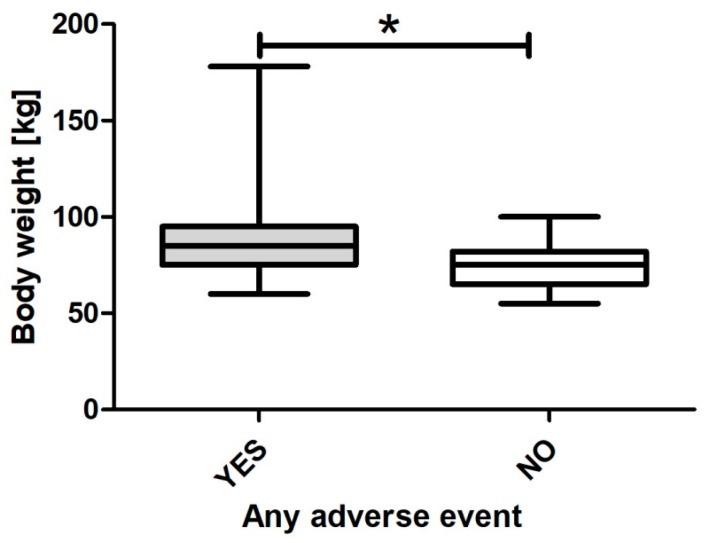
Body weight across any adverse event. * signifies statistically significant difference.

**Figure 3:**
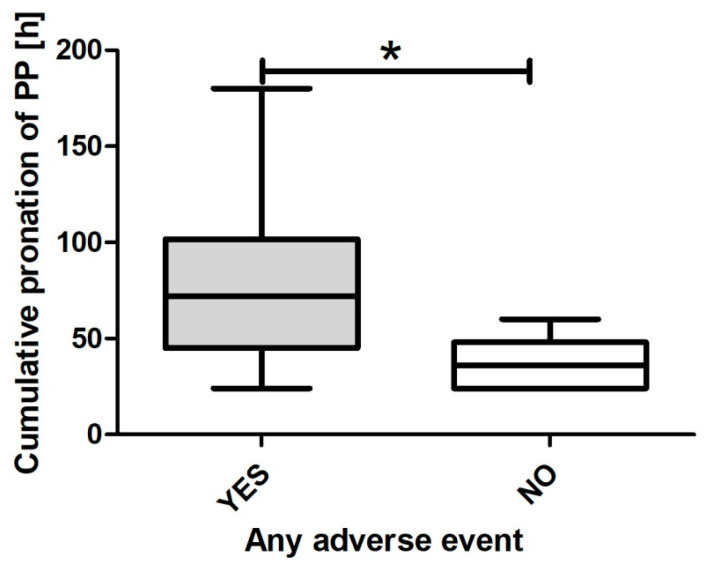
Cumulative duration of PP across any adverse event. * signifies statistically significant difference.

Relationships between body weight, age, and cumulative duration of PP itself were further assessed using generalized linear models. It was observed that weight (*p* = 0.021; β = 0.09 (CI95: 0.01–0.17), Figure 2) and the cumulative duration of PP (*p* = 0.005; β = 0.06 (CI95: 0.02–0.11), Figure 3) but not age (*p* = 0.782; β = −0.02 (CI95: −0.13–0.09)), were independently associated with any adverse event.

## 4. Discussion

PP is one of the few treatment options in the ICU supported with evidence from a positive large, prospective, interventional, randomized study [12]. It has demonstrated efficacy from the pre-pandemic era [13] and has been widely reported as successful during the COVID-19 pandemic [14,15,16]. PP is a cost-effective intervention that can be implemented without any (or minimal) additional medical equipment. The reduction in mortality is most pronounced in patients with more severe ARDS forms [17]. It was utilized in about one-third of patients with severe ARDS in the pre-pandemic era, and its use accelerated during the COVID-19 pandemic because of the increased number of patients [18]. Early initiation of PP with prolonged sessions (>16 h) has been shown to reduce mortality in patients with ADRS [13].

Our findings regarding the proportion of patients who underwent PP (36%) and their mortality (ICU mortality 37%, hospital mortality 47%) are roughly comparable to other studies [14,15,16,17,18]. However, we observed an unexpectedly high prevalence of unplanned removals of vascular catheters, drainage tubes, and endotracheal and tracheotomy tubes (118 unplanned removals in 66 patients, out of 100 patients who underwent PP). In comparison, other studies on PP where unplanned removals of medical devices were reported, the unplanned removal of arterial catheters was reported in 0–3.8% of patients (compared to 12.7% in our study) [19,20,21,22,23] and the unplanned removal of central venous catheters in 2.4% of patients (compared to 18.6% in our study) [19,20,21,22,23]. Similarly, unplanned removals of nasogastric tubes were reported at lower rates in other studies (6.1% compared to 29.6% in our study) [19,20,21,22,23]. The prevalence of unplanned removals in our study was even higher when compared to a general ICU patient population. In an 8-year pre-pandemic observational study [24] in a tertiary-level mixed ICU and post-surgery ICU, during which 10,514 patients were admitted to the ICU, 451 catheters, tubes, and drains were accidentally removed, mostly nasogastric tubes (270) and central venous and arterial catheters (92) but also endotracheal tubes (27), tracheostomy cannulas (1), and extra-corporeal membrane oxygenation cannulas (1). The ICUs were staffed exclusively by doctors and nurses who worked only in the ICU. Most removals occurred during the morning shift compared to afternoon or night shifts. They also observed that most removals were by patients themselves (368 out of 451 removals, or 81.6%), and the greatest number of removals occurred in patients with a Richmond agitation-sedation scale (RASS) score between 1 and 3 points (335 out of 451 removals, or 74.3%). In 61.4% of cases of removal, the patients were physically restrained at the time of accidental removal, highlighting the risk of unplanned removal of devices in patients with an altered mental state, which is common in the ICU [24]. In contrast to these settings, our patients were immobile, all patients were mechanically ventilated and intubated, and PP was used in all patients.

Regarding the unplanned removal of endotracheal or tracheal tubes, 16.1% of patients in our study required reintubation or repositioning of endotracheal or tracheal tubes, compared to 3.3% [25] and 3.8% [21] in other studies where PP was used. Despite the higher incidence of unplanned removals of catheters and tubes, the prevalence of cardiac arrest during ongoing PP in our study was lower compared to other reports (1.7% in our study compared to 6.7% in other studies) [13]. These differences can be partially explained by methodology—we defined unplanned removal as any removal within 24 h after supination following PP. While the removal of some catheters of tubes becomes clinically evident immediately (e.g., suffocation after removal of endotracheal tube), in some cases, the need for replacement becomes evident only following supination, and replacement can be postponed (e.g., kinking and partial occlusion of a central venous catheter). Additionally, we believe that the higher prevalence of unplanned removals in our case can be explained by the circumstances: one-third of nurses and one half of doctors working in the COVID ICU had previously worked in the ICU setting, while others have been recruited from other, non-ICU departments. Other authors have also reported on the increased frequency of adverse events and increased awareness of clinical teams of unsafe working environments during the COVID-19 pandemic. This was ascribed to a lack of personnel and the insufficient number and improvised nature of patient beds, difficulties in isolating a patient with COVID-19, and difficulties in the use of sedatives and other psychoactive medications in critically ill patients with COVID-19 [25]. Critical care clinicians’ experience regarding patient safety during the COVID-19 pandemic was one of more hazardous treatment conditions when compared to the pre-pandemic period, with most patient safety risks related to ventilator-related events [25].

Similarly, we recorded a higher prevalence of pressure ulcers compared to other authors—pressure ulcers developed in 79 patients (79%), with a great majority of patients developing facial and anterior thorax ulcers. The development of pressure ulcers is important because it has been associated with increased mortality in mechanically ventilated patients. This seems self-explanatory because the occurrence of pressure ulcers is more likely in higher-illness-severity patients. A regression analysis of a large observational patient cohort demonstrated increasing mortality with increasing severity of pressure injuries despite adjustment for those covariates [26,27]. The prevalence of pressure ulcers in other studies where PP was utilized ranged from 13% [22] to 42.9% [23]. In a pre-pandemic observational multicenter and multinational study [26] including ICU patients regardless of illness severity or PP, the prevalence of pressure ulcers was 26.6%. Nonmodifiable factors, such as older age, male sex, being underweight, admission due to emergency surgery, decreasing Braden scores, increasing ICU length of stay, presence of chronic obstructive pulmonary disease, immunodeficiency, renal replacement therapy, mechanical ventilation on ICU admission, and higher SAPS II score were independently associated with the development of pressure ulcers, with the strongest associations present with decreasing Braden scores and increasing length of stay. Hospitalization in low- or lower-middle-income economy ICUs was also associated with the development of pressure ulcers, possibly because of the limited availability of human resources, among other factors [26]. Other factors, such as the use of neuromuscular blocking agents and subsequent need for deep sedation might have also contributed to pressure ulcers in our setting. The decision to use PP was as per the treating physician, but it was perceived as a strategy to be used in higher-severity-of-illness patients. The rationale for a neuromuscular blockade was twofold—as a lung protective strategy along with lung-protective ventilation parameters [28] and as a safety strategy to prevent patient self-removal of catheters, tubes, and drains [29].

In our study, the main variables associated with an increased occurrence of bedsores were the length of time spent in PP (individual cycle and total time) and repetition of PP sessions [8]. Inadequate staffing and a different PP strategy could have contributed to greater prevalence of pressure ulcers in our study. PP was performed in 24 h sessions, while most other authors utilized around 16 h PP sessions. The optimal duration of PP sessions is uncertain [30], and we utilized longer sessions as a means to reduce the number of PP-related procedures in an understaffed ICU. Okin et al. [31] performed a multicenter retrospective study on 267 patients with COVID-19 requiring PP. The use of prolonged PP sessions (24 h or more) was associated with a significantly reduced number of PP sessions and with better 30- and 90-day survival compared to intermittent PP sessions (around 16 h). While there were no differences in the overall rate of PP-related complications, they reported increased rates of facial edema in patients who underwent prolonged PP sessions (15.3% vs. 6.4%) [31]. More frequent changes in body position from supine to prone (or vice versa) also requires increased doses of sedatives, muscle relaxants, and vasopressors, again increasing the need for medical staff [32]. Additionally, the »swimmer« position, the use of pillows, and the frequency of body position changes could have led to higher prevalence of pressure ulcers in our study. The position of the head, arms, and legs was changed once per shift (every 8 h), while in other studies, the position of the head, arms, and legs was usually changed every two hours [20,21,22].

Additional analysis of our patient cohort revealed that weight and the cumulative duration of PP were independently associated with the development of any adverse event. Binda et al. [23] reported that the cumulative duration of PP, and not weight, was independently associated with the acquisition of pressure ulcers; however, they utilized a different strategy with shorter (16 h) sessions and more frequent (every 2 h) changes in the position of the head, arms, and legs in the swimmer position. Girard et al. [7] performed an ancillary study of the PROSEVA study and observed that higher body mass index was independently associated with the acquisition of pressure ulcers. Higher BMI per se has also been associated with the greater likelihood of the occurrence of pressure ulcers in the ICU, and obese patients have a higher pressure ulcer score compared to normal weight patients. The association between obesity and severity of SARS-CoV-2 infection has also been documented [32].

We performed a single-center, retrospective study with inherent biases and limitations due to the design of the study. Data extraction was performed from a paper-based ICU in a setting where all procedures could not be adequately documented. Our definitions of PP-related adverse events were influenced by the ability to detect these events retrospectively. Despite this, we believe that important lessons should be learned from our study: first, local quality measurement and benchmarking data collections should be encouraged with the goal of improving patient outcomes; second, the duration and number of PP sessions should be weighed with regard to adverse events, especially in overweight patients; and third, personnel who perform prone positioning should be adequately educated.

## 5. Conclusions

We observed a significantly higher prevalence of adverse events, including both unplanned removals of catheters and tubes and pressure ulcers in our patient cohort compared to other studies. This disparity may be partially attributed to variations in methodology and partially to center-specific conditions during the outbreaks of COVID-19 (e.g., shortage of ICU-specialized staff, 24 h duration of PP sessions, positioning of the patient, etc.). We identified the cumulative duration of PP and higher body weight as the most important factors associated with the increased risk of adverse events. PP should be used with adverse events in mind, and appropriate ICU staff numbers and education are needed to ensure the appropriate quality and safety of PP.

## Figures and Tables

**Table 1:** Basic characteristics of patients who were included in the study (patients with COVID-19 and mechanical ventilation and PP).

Male Gender	60 (60%)
Age, years ± standard deviation	64.8 ± 9.1
ICU mortality	37 (37%)
Hospital mortality	47 (47%)

**Table 2:** Removal of vascular catheters, endotracheal and tracheal tubes, and other drainage tubes and catheters during PP.

Complications	n = 118
Removal CVC	15 (12.7%)
Removal arterial line	22 (18.6%)
Removal nasogastric tube	35 (29.6%)
Removal urinary catheter	17 (14.4%)
Removal dialysis catheter	0 (0%)
Reintubation or repositioning of tracheostomy tube during or immediately after supination	19 (16.1%)
Corneal injury	5 (4.2%)
Upper respiratory tract bleeding	2 (1.6%)
Brachial nerve injury	1 (0.8%)
CPR during pronation	2 (1.6%)

**Table 3:** Pressure ulcers associated with PP; distribution by location and stage.

Pressure Ulcers by Location	Number (%)	Stage I (%)	Stage II (%)	Stage III (%)	Stage IV (%)
Face	71 (38.5%)	0 (0%)	58 (73.4%)	13 (7.1%)	0 (0%)
Thorax	43 (23.3%)	0 (%)	40 (21.7%)	3 (1.6%)	0 (0%)
Extremities	28 (15.2%)	0 (0%)	28 (15.2%)	0 (0%)	0 (0%)
Anywhere anterior	42 (23%)	0 (0%)	42 (23%)	0 (0%)	0 (0%)

**Table 4:** Effect of PP session on stage of pressure ulcers.

Location and Stage of Pressure Ulcers	PP session 1	PP session 2	PP session 3	PP session 4	PP session 5
Face (n = 71)					
stage II stage III	15 (21.1%) 0 (0%)	19 (26.7%) 1 (1.4%)	20 (28.1%) 4 (5.6%)	4 (5.0%) 5 (7.0%)	0 (0%) 3 (4.2%)
Thorax (n = 43)					
stage II stage III	7 (16.2%) 0 (0%)	6 (13.9%) 1 (2.3%)	13 (30.2%) 2 (4.6%)	8 (18.6%) 0 (0%)	2 (4.6%) 1 (2.3%)
Extremities (n = 28)					
stage II stage III	4 (14.2%) 0 (0%)	4 (14.2%) 0 (0%)	10 (35.7%) 0 (0%)	7 (25.0%) 0 (0%)	3 (10.7%) 0 (0%)
Anywhere anterior (n = 42)					
stage II stage III	5 (11.9%) 0 (0%)	10 (23.8%) 0 (0%)	14(33.3%) 0 (0%)	9 (21.4%) 0 (0%)	3 (7.1%) 0 (0%)

**Table 5:** Association of the number of PP sessions with pressure ulcers and other adverse events.

	Session 1 (n = 24)	Session 2 (n = 39)	Session 3 (n = 25)	Session 4 (n = 9)	Session 5 (n = 3)
Location of pressure ulcers					
Face	15	20	24	9	3
Thorax	7	9	15	9	3
Extremities	4	3	11	7	3
Anywhere anterior	6	12	14	9	3
Other complications					
Removal CVC	2	2	6	4	1
Removal arterial line	2	8	7	4	1
Removal nasogastric tube	11	8	12	4	0
Removal urinary catheter	5	2	7	1	2
Removal dialysis catheter	0	0	0	0	0
Reintubation during or immediately after supination	3	*7*	*6*	*2*	*1*
Corneal injury	0	0	0	2	3
Upper respiratory tract bleeding	0	1	1	0	0
Brachial nerve injury	0	0	0	1	0
CPR during pronation	0	1	0	0	1

## Data Availability

Aggregated data are presented in the manuscript, and raw data are unavailable due to privacy restrictions.

## Public Involvement Statement

No public involvement in any aspect of this research.

## Guidelines and Standards Statement

This manuscript was drafted against the STROBE guidelines for observational studies.

## References

[1] Ghelichkhani P., Esmaeili M. (2020). Prone Position in Management of COVID-19 Patients; a Commentary. Arch. Acad. Emerg. Med..

[2] Gattinoni L., Tognoni G., Pesenti A., Taccone P., Mascheroni D., Labarta V., Malacrida R., Di Giulio P., Fumagalli R., Pelosi P. (2001). Effect of prone positioning on the survival of patients with acute respiratory failure. N. Engl. J. Med..

[3] Sud S., Friedrich J.O., Adhikari N.K.J., Taccone P., Mancebo J., Polli F., Latini R., Pesenti A., Curley M.A.Q., Fernandez R. (2014). Effect of prone positioning during mechanical ventilation on mortality among patients with acute respiratory distress syndrome: A systemaic review and meta-analysis. CMAJ.

[4] Fourie A., Ahtiala M., Black J., Hevia H., Coyer F., Gefen A., LeBlanc K., Smet S., Vollman K., Walsh Y. (2021). Skin damage prevention in the prone ventilated critically ill patient: A comprehensive review and gap analysis (PRONEtect study). J. Tissue Viability.

[5] Koulouras V., Papathanakos G., Papathanasiou A., Nakos G. (2016). Efficacy of prone position in acute respiratory distress syndrome patients: A pathophysiology-based review. World J. Crit. Care Med..

[6] Munshi L., Del Sorbo L., Adhikari N.K.J., Hodgson C.L., Wunsch H., Meade M.O., Uleryk E., Mancebo J., Pesenti A., Ranieri V.M. (2017). Prone Position for Acute Respiratory Distress Syndrome. A Systematic Review and Meta-Analysis. Ann. Am. Thorac..

[7] Girard R., Baboi L., Ayzac L., Richard J.C., Guérin C. (2014). The impact of patient positioning on pressure ulcers in patients with severe ARDS: Results from a multicentre randomised controlled trial on prone positioning. Intensive Care Med..

[8] Lucchini A., Bambi S., Mattiussi E., Elli S., Villa L., Bondi H., Rona R., Fumagalli R., Foti G. (2020). Prone Position in Acute Respiratory Distress Syndrome Patients: A Retrospective Analysis of Complications. Dimens. Crit. Care Nurs..

[9] Liaw A., Wiener M. (2002). Classification and Regression by randomForest. R. News.

[10] Guyon I., Weston J., Barnhill S., Vapnik V. (2002). Gene Selection for Cancer Classification using Support Vector Machines. Mach. Learn..

[11] Robin X., Turck N., Hainard A., Tiberti N., Lisacek F., Sanchez J.C., Müller M. (2011). pROC: An open-source package for R and S+ to analyze and compare ROC curves. BMC Bioinform..

[12] Laffey J.G., Kavanagh B.P. (2018). Negative trials in critical care: Why most research is probably wrong. Lancet Respir. Med..

[13] Guérin J., Reignier J.C., Richard P., Beuret A., Gacouin T., Boulain T., Mercier E., Badet M., Mercat A., Baudin O. (2013). Prone positioning in severe acute respiratory distress syndrome. N. Engl. J. Med..

[14] Langer T., Brioni M., Guzzardella A., Carlesso E., Cabrini L., Castelli G., Dalla Corte F., de Robertis E., Favarato M., Forastieri A. (2021). Prone position in intubated, mechanically ventilated patients with COVID-19: A multi-centric study of more than 1000 patients. Crit Care.

[15] Camporota L., Sanderson B., Chiumello D., Terzi N., Argaud L., Rimmelé T., Metour R., Verstraete A., Cour M., Bohé J. (2022). Prone Position in COVID-19 and -COVID-19 Acute Respiratory Distress Syndrome: An International Multicenter Observational Comparative Study. Crit. Care Med..

[16] Engerström L., Thermaenius J., Mårtensson J., Oldner A., Petersson J., Kåhlin J., Larsson E. (2022). Prevalence and impact of early prone position on 30-day mortality in mechanically ventilated patients with COVID: A nationwide cohort study. Crit. Care.

[17] Bloomfield R., Noble D.W., Sudlow A. (2015). Prone position for acute respiratory failure in adults. Cochrane Database Syst. Rev..

[18] Chiumello D., Coppola S., Froio S. (2018). Prone position in ARDS: A simple maneuver still underused. Intensive Care Med..

[19] Ibarra G., Rivera A., Fernandez-Ibarburu B., Lorca-García C., Garcia-Ruano A. (2021). Prone position pressure sores in the COVID-19 pandemic: The Madrid Experience. J. Plast. Reconstr. Aesthet. Surg..

[20] Page D.B., Vijaykumar K., Russell D.W., Gandotra S., Chiles J.W., Whitson M.R., Dransfield M.T. (2022). Prolonged Prone Positioning for COVID-19-induced Acute Respiratory Distress Syndrome: A Randomized Pilot Clinical Trial. Ann. Am. Thorac. Soc..

[21] Sastre J.A., López J., Vaquero-Roncero L.M., Sánchez-Barrado M.E., Martín-Moreno M.A., Arribas P., Hernandez A., Garrido-Gallego I., Sanchez-Hernandez M.V. (2021). Clinical features and respiratory pathophysiology of COVID-19 patients ventilated in the prone position: A cohort study. Anaesthesiol. Intensive Ther..

[22] Rodríguez-Huerta M.D., Díez-Fernández A., Rodríguez-Alonso M.J., Robles-González M., Martín-Rodríguez M., González-García A. (2022). Nursing care and prevalence of adverse events in prone position: Characteristics of mechanically ventilated patients with severe SARS-CoV-2 pulmonary infection. Nurs. Crit. Care.

[23] Binda F., Galazzi A., Marelli F., Gambazza S., Villa L., Vinci E., Adamini I., Laquintana D. (2021). Complications of prone positioning in patients with COVID-19: A cross-sectional study. Intensive Crit. Care Nurs..

[24] Galazzi A., Adamini I., Consonni D., Roselli P., Rancati D., Ghilardi G., Greco G., Salinaro G., Laquintana D. (2019). Accidental removal of devices in intensive care unit: An eight-year observational study. Intensive Crit. Care Nurs..

[25] Rosen A., Carter D., Applebaum J.R., Southern W.N., Brodie D., Schwartz J., Cornelius T., Shelton R.C., Yip N.H., Pincus H.A. (2022). Critical Care Clinicians’ Experiences of Patient Safety During the COVID-19 Pandemic. J. Patient Saf..

[26] Labeau S.O., Afonso E., Benbenishty J., Blackwood B., Boulanger C., Brett S.J., Calvino-Gunther S., Chaboyer W., Coyer F., Deschepper M. (2021). Prevalence, associated factors and outcomes of pressure injuries in adult intensive care unit patients: The DecubICUs study. Intensive Care Med..

[27] Manzano F., Pérez-Pérez A.M., Martínez-Ruiz S., Garrido-Colmenero C., Roldan D., Del Mar Jiménez-Quintana M., Sánchez-Cantalejo E., Colmenero M. (2014). Hospital-acquired pressure ulcers and risk of hospital mortality in intensive care patients on mechanical ventilation. J. Eval. Clin. Pract..

[28] Papazian L., Forel J.-M., Gacouin A., Penot-Ragon C., Perrin G., Loundou A., Jaber S., Arnal J.-M., Perez D., Seghboyan J.M. (2010). Neuromuscular blockers in early acute respiratory distress syndrome. N. Engl. J. Med..

[29] Iavarone I.G., Al-Husinat L., Vélez-Páez J.L., Robba C., Leme Silva P., Rocco P.R.M., Battaglini D. (2024). Management of Neuromuscular Blocking Agents in Critically Ill Patients with Lung Diseases. J. Clin. Med..

[30] Guérin C., Albert R.K., Beitler J., Gattinoni L., Jaber S., Marini J.J., Munshi L., Papazian L., Pesenti A., Vieillard-Baron A. (2020). Prone position in ARDS patients: Why, when, how and for whom. Intensive Care Med..

[31] Okin D., Huang C.Y., Alba G.A., Jesudasen S.J., Dandawate N.A., Gavralidis A., Chang L., Moin E.E., Ahmad I., Witkin A.S. (2023). Prolonged Prone Position Ventilation Is Associated with Reduced Mortality in Intubated COVID-19. Patients. Chest.

[32] Simonnet A., Chetboun M., Poissy J., Raverdy V., Noulette J., Duhamel A., Labreuche J., Mathieu D., Pattou F., Jourdain M. (2020). High Prevalence of Obesity in Severe Acute Respiratory Syndrome Coronavirus-2 (SARS-CoV-2) Requiring Invasive Mechanical Ventilation. Obesity.

